# Steel Corrosion Evaluation of Basalt Fiber RPC Affected by Crack and Steel-Concrete Interface Damage Using Electrochemical Methods

**DOI:** 10.3390/s20185027

**Published:** 2020-09-04

**Authors:** Hanbing Liu, Xiang Lyu, Yuwei Zhang, Guobao Luo, Wenjun Li

**Affiliations:** College of Transportation, Jilin University, Changchun 130025, China; lhb@jlu.edu.cn (H.L.); lvxiang18@mails.jlu.edu.cn (X.L.); luogb17@mails.jlu.edu.cn (G.L.); wenjun18@mails.jlu.edu.cn (W.L.)

**Keywords:** reactive powder concrete, basalt fiber, electrochemical, crack, steel-concrete interface damage

## Abstract

Basalt fiber (BF) is a new anti-corrosion and environmentally friendly material, which is expected to delay the corrosion process of steel bars and improve the durability of reinforced reactive powder concrete (RPC). The electrochemical method is a nondestructive testing and real-time monitoring technique used to characterize the corrosion behaviors of steel bars embedded in concrete structures. In this paper, the electrochemical technique was employed to evaluate the corrosion of steel bars embedded in basalt fiber modified reactive powder concrete (BFRPC). Besides, crack and steel-concrete interface damage (SCID) were considered as typical factors that affect steel corrosion in concrete. Thus, both reinforced fiber-free RPC and BFRPC specimens with crack and SCID were prepared for evaluating the steel corrosion behaviors by electrochemical methods. The results revealed that both crack and SCID would aggravate the steel corrosion, and the crack was the major factor that affects the corrosion process. Moreover, the excellent compactness of BFRPC and the bridging action of BF could effectively prevent the concrete cracking and steel corrosion process of concrete. Using reinforced BFRPC instead of ordinary concrete in practical projects could greatly extend the service life of steel bars.

## 1. Introduction

Steel corrosion is the most common and important factor of durability damage, which greatly reduces the service life of ordinary concrete structures [1,2]. Therefore, the research of steel corrosion in concrete is of great significance to the sustainability of the concrete structure. In ordinary concrete, the strength of the interfacial transition zone between coarse aggregate and mortar is weak due to the difference between the elastic modulus, linear expansion coefficient, and other properties of coarse aggregate and mortar. Adverse matters (such as chloride ions) are easy to penetrate in concrete and cause steel corrosion, which further leads to premature concrete destruction. To avoid this natural defect, a new type of cement-based material, reactive powder concrete (RPC), with ultra-high strength, high durability, and high-temperature adaptability appeared [3]. In RPC materials, coarse aggregates were replaced by fine quartz sand, which optimized particle gradation, improved uniformity, and controlled internal defects [4,5]. Therefore, RPC is expected to be widely used in bridges and building structures in adverse environments, such as seasonally frozen regions and oceans due to its excellent properties [6,7]. Besides, related studies [8,9] showed that fiber can improve RPC performance. However, steel fiber is prone to rust, carbon fiber has a high price, and glass fiber has poor alkali resistance. Therefore, these fibers are not conducive to the promotion and application of RPC [10,11,12]. Basalt Fiber (BF) is a new type of inorganic fiber with natural compatibility and excellent mechanical properties. It is made of pure natural basalt ore by the melt drawing process. Thus, BF is an eco-friendly material that is free from pollution in the production process. Moreover, the density of BF is similar to that of cement concrete and mortar, so BF can be evenly distributed in concrete. Previous studies [13,14,15,16,17,18] have also illustrated that BF could improve the toughness and other properties of concrete structures. However, there are few studies on the long-term durability of steel bars embedded in basalt fiber reactive powder concrete (BFRPC) in an adverse environment.

Chloride penetration is an important factor that causes steel corrosion. In cold regions, chloride is commonly used to melt snow on the surface of reinforced concrete bridges in winter. Therefore, the chloride ions dissolved in the snow will gradually penetrate the concrete. Gode et al. [19] found that de-icing salt leaks into bridge concrete from expansion joints and waterproof film, which seriously reduces the service life of bridge structures built with concrete. Vu et al. [20] found that the statistical mean value of chlorine accumulation concentration on the surface of reinforced concrete bridges under the action of deicing salt in cold regions was 3.5 kg/m^3^. Besides, cracks and steel-concrete interface damage (SCID) would inevitably occur during the long-term service of reinforced concrete structures, which would accelerate the steel corrosion [21,22]. Hay et al. [23] studied the influence of artificial cracks and SCID on the corrosion of ordinary concrete steel bars and reported that the existence of cracks increased the corrosion rate of steel bars, and the SCID further intensified the corrosion. Chen et al. [24] found that porous bands at the steel-concrete interface in making concrete can significantly affect the vacuum process of concrete. In this paper, according to the deicing salt environment of bridge constructions in cold regions, reinforced BFRPC specimens were immersed in a chloride solution for evaluating the steel corrosion behavior. Moreover, crack and SCID were selected as two damage conditions of in-service reinforced concrete materials.

At present, the corrosion testing methods of steel bars embedded in concrete include the nondestructive testing method, physical method, and chemical method. The nondestructive testing method mainly uses acoustic, optical, electrical, and other technical means to diagnose the defects of materials [25,26,27]. The physical method is to use physical or mechanical means (optical fiber sensor, resistance probe technology, magnetic resistance probe technology, etc.) to monitor and analyze the corrosion process [28,29]. However, the physical method is more complicated, and the sensor has a shorter life and higher price. The chemical method means to judge the corrosion status of steel bars indirectly according to the content of chemical composition in concrete protective layer (PH value of pore fluid, chloride ion content on steel bar surface, etc.) [30]. However, the chemical method is required to destroy the structure, and the location, speed, and state of corrosion cannot be determined. As a new testing technology in recent years, the electrochemical method has the advantages of simple equipment, low cost, high accuracy, wide range, and high sensitivity [31]. Therefore, electrochemical methods were used to evaluate the corrosion of steel bars embedded in concrete. In recent years, electrochemical methods have been widely used in the monitoring and evaluation of steel corrosion. Monzon et al. [32] studied the corrosion rate of reinforced concrete by linear polarization method and electrochemical impedance spectroscopy (EIS) and analyzed various electrochemical parameters by partial least squares. Duprat et al. [33] studied the statistical quantification of the Tafel coefficient of steel bars in concrete. Chang et al. [34] evaluated the corrosion rate and Tafel parameters of three types of steel bars in concrete, new steel bars, and corroded steel bars in seawater. Soleymani et al. [35] compared different corrosion measurement methods and verified the differences between the test results of various electrochemical methods. Ghafari et al. [36] added nano-silica into ultra-high-performance concrete to reduce the corrosion rate of steel bars and adopted the Tafel diagram, linear polarization resistance, and other multi-step potential dynamic polarization technologies to determine the corrosion rate of specimens through accelerated corrosion test. Reou et al. [37] et al. studied the corrosion of embedded steel in concrete by using electrochemical technology, and found that the electrochemical test results can not only indicate whether corrosion has begun, but also monitor the development of corrosion.

Although there are lots of laboratory studies about the mechanical properties of BFRPC material, the durability of reinforced BFRPC under the effect of crack and SCID is not clear. The purpose of this paper is to in-advance study the corrosion characteristics of steel bars under the effects of common damages of reinforced BFRPC so that this material can be widely used in coastal and seasonal frozen regions. In this paper, the influence of crack and SCID on the corrosion of steel bars embedded in BFRPC was studied by electrochemical methods, which aimed to lay a foundation for the popularization and application of reinforced BFRPC in practical engineering.

## 2. Electrochemical Method

Tafel potentiodynamic polarization (TPP) and Electrochemical impedance spectrum (EIS) measurements were utilized in this study for evaluating the steel corrosion behavior.

### 2.1. TPP Measurement

The polarization curve refers to the relationship between current density and electrode potential, In addition, the polarization curve is one of the most basic and important methods to analyze the electrode process dynamics. The polarization curve includes a strong polarization region (Tafel region), a weak polarization region, and a linear polarization region. The Tafel region can obtain reliable information about the anodic and cathodic reactions in the corrosion process, and the Tafel curve can reveal the mechanism of working electrode corrosion. The TPP measurement is to scan the Tafel region with the potentiodynamic scanning and obtain the Tafel curve.

The relationship between electric potential E (V) and current density logarithm I (A/cm^2^) in the Tafel polarization curve is shown in Equation (1).

(1) $$I=i_{\mathrm{corr}}\left\{\exp\left[\frac{2.303\left(E-E_{\mathrm{corr}}\right)}{b_{a}}\right]-\exp\left[\frac{2.303\left(E_{\mathrm{corr}}-E\right)}{b_{c}}\right]\right\}$$

where b_a_ and b_c_ are the anodic Tafel slope and cathodic Tafel slope, respectively, unit: mV/Decade; E_corr_ is the corrosion potential compared with the saturated calomel electrode (SCE), unit: V; i_corr_ is the self-corrosion current density, unit: μA/cm^2^.

**Figure 1:**
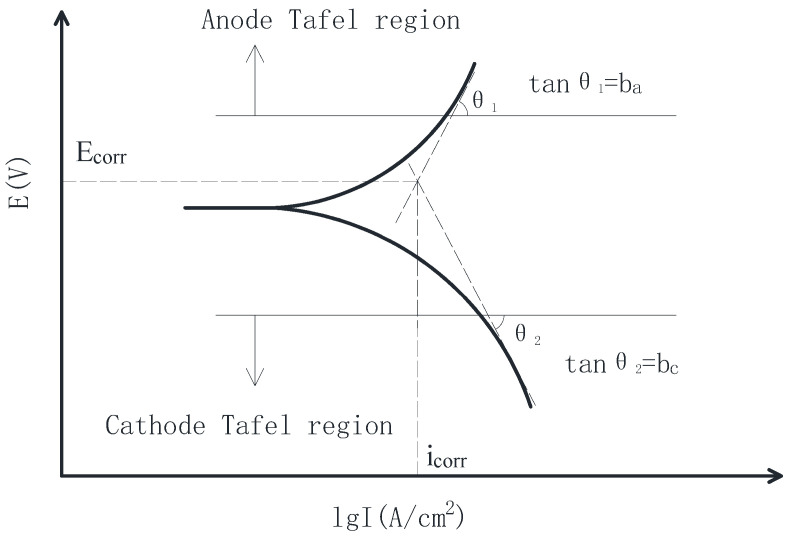
Tafel potentiodynamic polarization (TPP) curve fitting.

The electrochemical parameter values in Equation (1) can be obtained by the extrapolation method of the Tafel curve. The fitting of the TPP curve is shown in Figure 1.

As can be seen from Figure 1, the Tafel curve is divided into two sections: the upper Tafel curve is the anode Tafel curve, and the lower Tafel curve is the cathode Tafel curve. The straight lines fitted by the anode Tafel curve and cathode Tafel curves will intersect at a point whose coordinate values are corrosion potential (E_corr_) and self-corrosion current density (i_corr_), respectively. The slope of the fitted straight line is b_a_ and b_c_, respectively.

In this paper, the TPP measurement range was between −250 mV and 250 mV of open circuit potential, and the measurement scanning rate was set to 3 mV/s.

### 2.2. EIS Measurement

EIS is repeatable and non-destructive. The impedance changes with frequency. The impedance is closer to a different device at different frequencies, such as a resistor or capacitor. By using different equivalent elements to fit the EIS curve, the corresponding equivalent circuit diagram and the parameters of the equivalent elements can be obtained.

**Figure 2:**
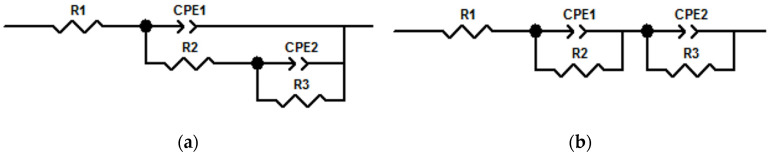
Equivalent circuit diagram: (**a**) no prefabricated crack specimens, such as reactive powder concrete (RPC) and basalt fiber reactive powder concrete (BFRPC); (**b**) Prefabricated crack specimens, such as RPC-Crack (C), RPC-C- steel-concrete interface damage (SCID), BFRPC-C, and BFRPC-C-SCID.

In this paper, EIS measurement was carried out for each steel bar in RPC and BFRPC specimens. The frequency range was 0.01–10^5^ Hz, and the AC amplitude was 10 mV. EIS could be divided into two types according to whether the specimen contains a prefabricated crack, as shown in Figure 2.

In Figure 2, R is an equivalent resistance of electrical components. CPE is a normal phase angle element, which is generally equivalent to a capacitor. R_1_ is electrolyte resistance, CPE_1_ is concrete capacitance, R_2_ is concrete resistance, and CPE_2_ is double-layer capacitance on the surface of the steel bars. R_3_ is the resistance of steel bars in concrete. The two equivalent circuit diagrams in Figure 2 are composed of three equivalent resistors and two constant phase angle elements. However, due to the different combinations of these equivalent elements, the impedances of two equivalent circuit diagrams are different. The corresponding impedances in Figure 2a,b are shown in Equations (2) and (3), respectively.

(2) $$Z=R_{1}+\frac{1}{\frac{1}{Z_{CPE_{1}}}+\frac{1}{R_{2}+\frac{1}{\frac{1}{Z_{CPE_{2}}}+\frac{1}{R_{3}}}}}$$

(3) $$Z=R_{1}+\frac{1}{\frac{1}{Z_{CPE_{1}}}+\frac{1}{R_{2}}}+\frac{1}{\frac{1}{Z_{CPE_{2}}}+\frac{1}{R_{3}}}$$

The specific values of each device in the impedance spectrum were obtained by fitting the experimental values and EIS. Then the steel resistance could be accurately fitted and the corrosion of steel could be evaluated.

## 3. Experimental Details

### 3.1. Materials

**Figure 3:**
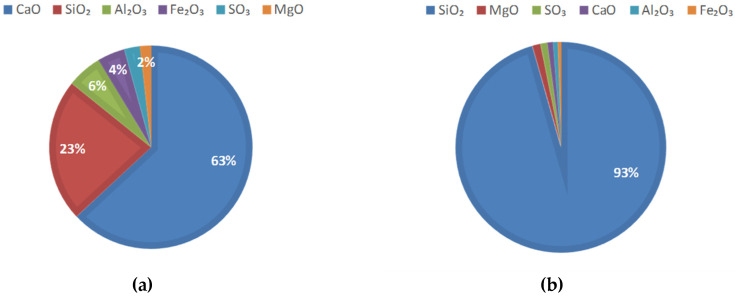
Chemical composition of cementing materials: (**a**) cement; (**b**) silica fume.

The materials required for BFRPC are quartz sand, quartz powder, silica fume, cement, water, water reducer, and basalt fiber. The main component of quartz sand is silicon dioxide, with a content of more than 99%. The quartz sand in this paper is produced by Zhenxing Quartz Sand Factory (Luoyang, China). 20–40 mesh, 40–70 mesh, and 80–120 mesh quartz sand are mixed in a 2:2:1 ratio for experimental sand. Experimental sand is sieved through 0.6 mm, 0.3 mm, and 0.15 mm square holes for later use. The size of quartz powder is 400 mesh. The mass fraction of silica fume is 93.3% and the specific surface area is 18100 m^2^/kg. The P.O 42.5 cement produced by Jilin Yatai Cement Co., Ltd. (Changchun, China) is used. The basic properties of basalt fibers are shown in Table 1. The chemical composition of silica fume and cement is shown in Figure 3. Besides, Q235 hot-rolled round steel bars with a diameter of 8 mm and a length of 380 mm were used, and the surface area of each steel bar is 96.56 cm^2^.

The optimal mix proportion was determined by the response surface method, according to our previous study [38], and this mix proportion was applied in this paper. Mix proportions of RPC and BFRPC materials were shown in Table 2.

### 3.2. Specimen Preparation

Three types of specimens were made, respectively, and each specimen has different conditions, as shown in Table 3. During the test, fiber-free RPC was used as the control group.

**Figure 4:**
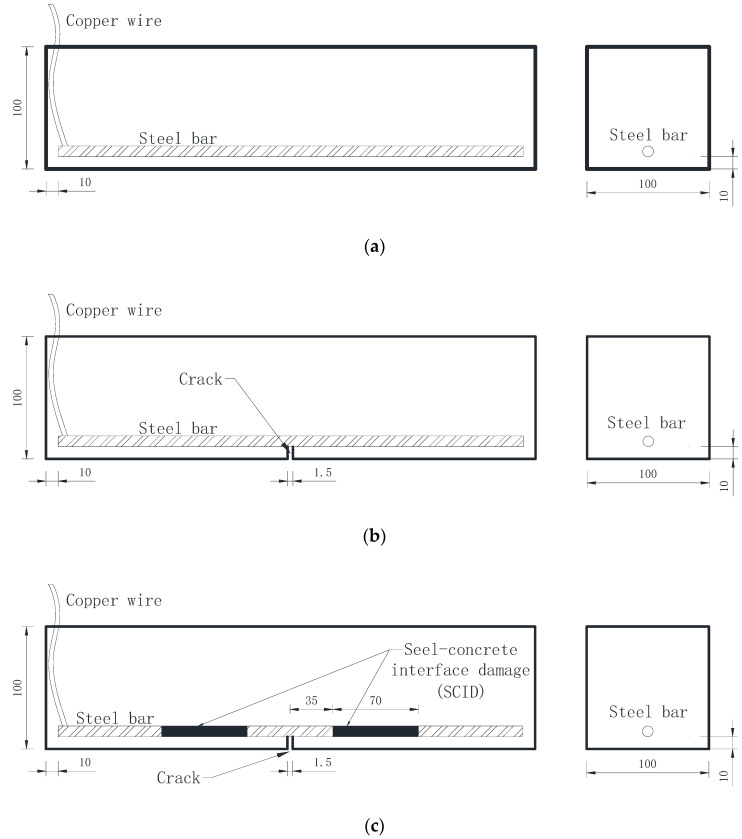
Schematic diagram of front and side views of specimen: (**a**) RPC and BFRPC; (**b**) RPC-C and BFRPC-C; (**c**) RPC-C-SCID and BFRPC-C-SCID.

To simulate the actual condition of the steel bar in the beam, specimens of 400 mm × 100 mm × 100 mm were prepared. The steel bar was placed at the bottom of the specimen with a protective thickness of 10 cm. Crack and SCID were treated as the typical deteriorated factor and applied for breaking the specimens of RPC and BFRPC in the preparation process. The schematic diagrams include the length and location of the steel bars and SCID inside each type of specimen are shown in Figure 4.

The cracks in Figure 4b,c were created by prepositioning. Prepositioning usually involves inserting a thin sheet of copper into the concrete and removing it before the concrete is fully hardened. However, cracks made by this method are easy to be filled with surrounding materials in the subsequent curing process, which will affect the accuracy of test results. In this paper, a wood sheet was used instead of a copper sheet. After the specimen was cured and molded, the wood sheet was taken out, which could avoid the test error caused by the preset copper sheet. The SCID in Figure 4 was achieved by wrapping filter paper on the surface of the steel bar.

### 3.3. Test Procedure

**Figure 5:**
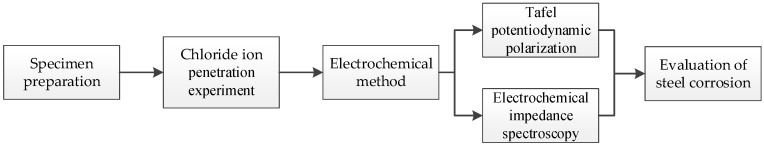
Experimental procedure.

The test process was divided into the following steps: Firstly, BF was added to RPC to prepare BFRPC. Then, crack and SCID were treated as the typical deteriorated factor and applied for breaking the specimens. Next, the specimens were cured in steam at 90 °C for 48 h. In order to analyze the applicability and the steel corrosion behaviors of reinforced BFRPC in the seasonal frozen regions, the specimens were placed in 5 wt.% NaCl solution for chloride ion penetration experiment. The chloride ion penetration experiment was performed in one cycle every 7 days, and a total of four cycles were needed. At the end of each cycle, electrochemical methods were used to monitor and evaluate the corrosion of the steel bar in each specimen. The experimental procedure is shown in Figure 5.

### 3.4. Electrochemical Test

**Figure 6:**
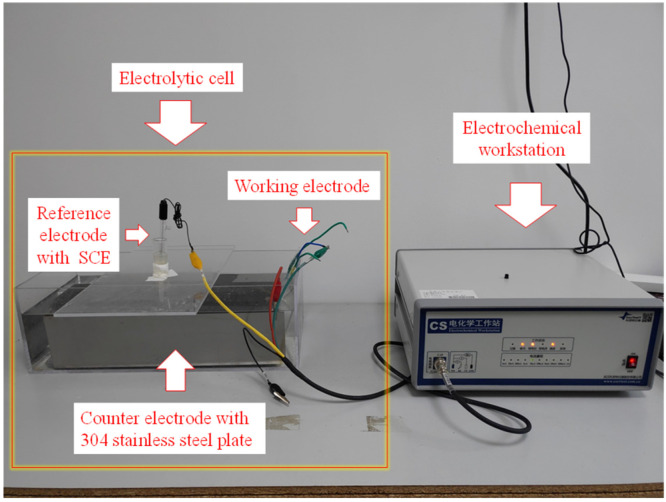
Electrochemical experimental setup.

A three-electrode system is commonly used in electrochemical testing. In this paper, CS350H electrochemical workstation produced by Coster Co., Ltd. (Wuhan, China) was selected to conduct electrochemical testing with a three-electrode system. In the three-electrode system, the measured system contained the working electrode (WE), reference electrode (RE), and counter electrode (CE). The steel bars welded with copper wires are treated as WE. RE consists of SCE, saturated potassium chloride, agar, and a Luggin capillary. A 304 stainless steel plate with 400 mm × 100 mm × 1 mm was adopted as CE. The electrolyte used in this study is 3.5 wt% NaCl solution. The electrochemical experimental setup is shown in Figure 6.

## 4. Result and Discussion

### 4.1. TPP Result

#### 4.1.1. Tafel Curve Analysis

**Figure 7:**
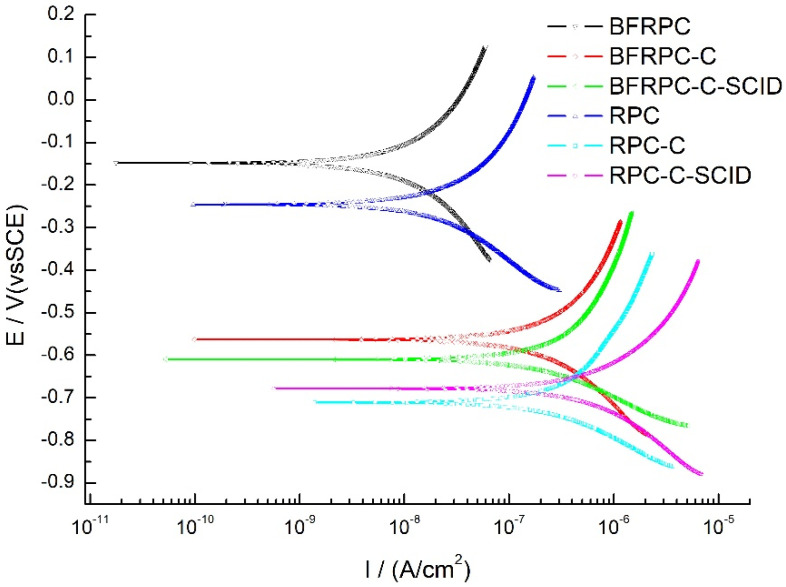
Tafel curves of all specimens (chloride ion penetration time: 7d).

Specimens with a chloride ion penetration time of 7 days were selected to analyze the influence of BF, crack, and SCID on the corrosion of steel bars. The TPP curve of all specimens could be obtained by TPP measurement, and the test results were shown in Figure 7.

Generally, when the E_corr_ was less than −0.275 V vs. SCE, there was a greater than 90% probability that steel bars in concrete structures were corroding [39]. Qualitatively, TPP curve moving in the negative direction of the Y-axis indicates that the corrosion resistance of the steel bar becomes poor. As can be seen from Figure 7, compared with the BFRPC and RPC specimens, the E_corr_ of the BFRPC-C, BFRPC-C-SCID, RPC-C and RPC-C-SCID specimens move in a negative direction of the Y-axis and the E_corr_ value is significantly less than −0.275 V, which indicates that the cracks weaken the corrosion resistance of the steel bars embedded in BFRPC-C, BFRPC-C-SCID, RPC-C, and RPC-C-SCID. Besides, under the same condition, the E_corr_ of RPC specimen is more inclined to the negative direction of the Y-axis than that of the BFRPC specimen, which indicates that the corrosion resistance of steel bars in RPC specimen is weaker, and BF can increase the corrosion resistance of steel bars in BFRPC.

To quantify the corrosion index of steel bar in more detail, Tafel curve parameters of steel bars could be obtained by using the fitting method shown in Figure 1. Table 4 shows the electrochemical parameters obtained by fitting each TPP curve in Figure 7, where b_a_ is the anode Tafel slope, b_c_ is the cathode Tafel slope, i_corr_ is the corrosion current density of steel bars, and E_corr_ is the corrosion potential of steel bars vs. SCE.

Related research showed that an active corrosion activity was taking place when the i_corr_ greater than 0.2μ/cm^2^ [40]. Table 4 shows that the E_corr_ of the steel bars is BFRPC > RPC > threshold value (−0.275 V) > BFRPC-C > BFRPC-C-SCID > RPC-C-SCID > RPC-C in order from largest to smallest. Moreover, the i_corr_ of steel bars is RPC-C-SCID > BFRPC-C > RPC-C > BFRPC-C-SCID > threshold value (0.2μA/cm^2^) > BFRPC > RPC in order from largest to smallest. This indicates that the steel bars embedded in BFRPC and RPC in Figure 7 have no corrosion, while the steel bars embedded in BFRPC-C, BFRPC-C-SCID, RPC-C, and RPC-C-SCID have an obvious corrosion activity. Besides, it can be seen that the value of E_corr_ and i_corr_ have an excellent reflection on whether the steel bars corrosion occurs. Therefore, E_corr_ and i_corr_ were used for a detailed analysis of all test data in this paper.

#### 4.1.2. Corrosion Potential

**Figure 8:**
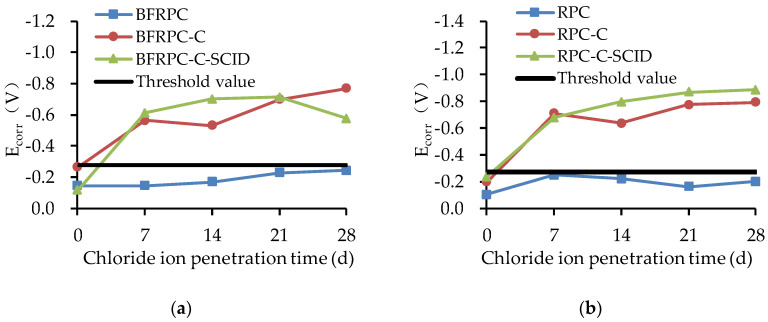
Corrosion potentials (E_corr_) for: (**a**) BFRPC specimens; (**b**) RPC specimens.

The E_corr_ results of the steel bars embedded in the BFRPC specimens and RPC specimens are given in Figure 8. The threshold value (−0.275 V) for the E_corr_ of steel bars is set to better distinguish whether steel bars are corroded or not.

It can be seen from Figure 8 that the changes of E_corr_ in BFRPC specimens and RPC specimens are highly consistent. Figure 8 shows that the E_corr_ of steel bars in BFRPC and RPC without cracks is greater than the threshold value (−0.275 V) within 7–28d of chloride ion penetration, whereas the E_corr_ of steel bars in BFRPC-C, BFRPC-C-SCID, RPC-C, and RPC-C-SCID with cracks is less than the threshold value (−0.275 V), within 7–28d of chloride ion penetration. This indicates that the crack has a great influence on the E_corr_. There is no corrosion of steel bars in concrete when there is no crack, while there is corrosion of steel bars in concrete when there is crack. This is because BFRPC and RPC used quartz sand instead of coarse aggregate in ordinary concrete, which improved the compactness between particles and prevented direct penetration of chloride ion into the steel bars layer. However, the existence of cracks provided a convenient channel for the chloride ion, so that the concentration of chloride ion on the surface of the steel bars reached the concentration of corrosion, and then caused the corrosion of the steel bars.

#### 4.1.3. Self-Corrosion Current Density

**Figure 9:**
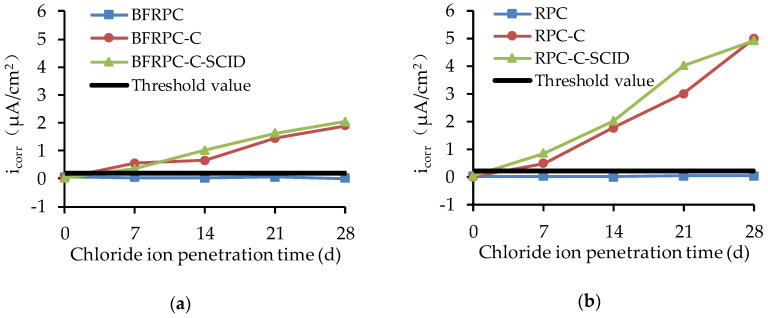
Self-corrosive current density (i_corr_) for: (**a**) BFRPC specimens; (**b**) RPC specimens.

The i_corr_ results of the steel bars embedded in the BFRPC specimens and RPC specimens are shown in Figure 9. The threshold value (0.2 μA/cm^2^) for the i_corr_ of steel bars is set so that it is better to identify whether there is active corrosion on the surface of steel bars. Furthermore, the larger i_corr_ is, the faster the corrosion rate of the steel bar is. Relevant studies [41,42] have shown that chloride ion penetration would cause steel corrosion in intact ordinary concrete. However, Figure 9 shows that the i_corr_ of the steel bars in BFRPC and RPC remains unchanged with the increase of chloride ion penetration time and the i_corr_ value is less than the threshold value (0.2 μA/cm^2^). This indicates that the steel bars in BFRPC and RPC will not corrode under chloride ion penetration, which is consistent with the result of E_corr_. The i_corr_ of steel bars in BFRPC-C, BFRPC-C-SCID, RPC-C, and RPC-C-SCID increases with the increase of chloride ion penetration time. Moreover, compared with BFRPC-C and RPC-C, specimens with SCID (BFRPC-C-SCID and RPC-C-SCID) have higher i_corr_. This can be concluded that both cracks and SCID will increase the corrosion rate of steel bars.

### 4.2. EIS Result

#### 4.2.1. EIS Analysis

**Figure 10:**
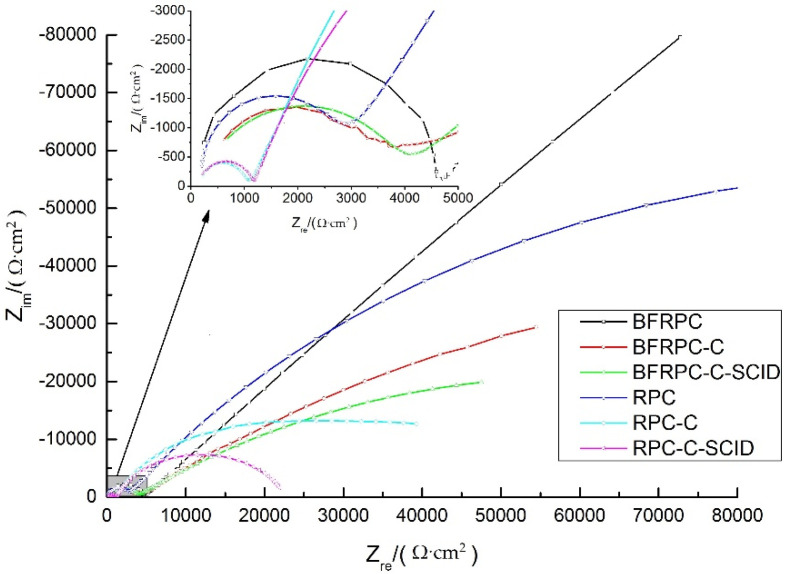
Nyquist curves of steel bars embedded in the specimens (chloride ion penetration time: 28d).

The Nyquist in Figure 10 were used to demonstrate the EIS results of steel bars embedded in the specimens. In Figure 10, the influences of BF, crack and SCID on the resistance of steel bars were analyzed by selecting data of the specimens with a chloride ion penetration time of 28 days. When the resistance of steel bars decreases gradually, the corrosion degree of steel bars increases gradually.

It can be seen from the qualitative analysis of Figure 10 that the Nyquist curves of different specimens contain two circular arcs. The first circular arc often called a high-frequency circular arc which represents an electrochemical reaction that occurs between the solid and liquid phases in the concrete matrix. Moreover, the second circular arc is called the low-frequency circular arc which represents the electrochemical reaction that occurs between the concrete matrix and the steel bars electrode.

To explain the obtained Nyquist curves results in Figure 10, electrochemical parameters are obtained by fitting the equivalent circuit diagram in Figure 2, and the results are shown in Table 5.

Table 5 shows that the steel bars in RPC have little difference in solution resistance R_1_, indicating that the properties of the electrolyte are relatively stable and will not change significantly during the test. Besides, it can be seen from Table 5 that the concrete resistance R_2_ of the BFRPC specimens is greater than that of the RPC specimens. This indicates that BF increased the resistance of the BFRPC matrix. Meanwhile, the R_3_ of the steel bars is BFRPC > RPC > BFRPC-C > BFRPC-C-SCID > RPC-C > RPC-C-SCID in order from largest to smallest. This showed that both cracks and SCID would cause steel resistance to decrease. In addition, the addition of BF reduces the corrosion degree of the steel bars. BF makes the connection of each part of RPC more closely and inhibits the further aggravation of the corrosion process. Therefore, BF can effectively control the corrosion rate of steel bars, reduce the corrosion degree of steel bars, and extend the service life of steel bars.

#### 4.2.2. Resistance

**Figure 11:**
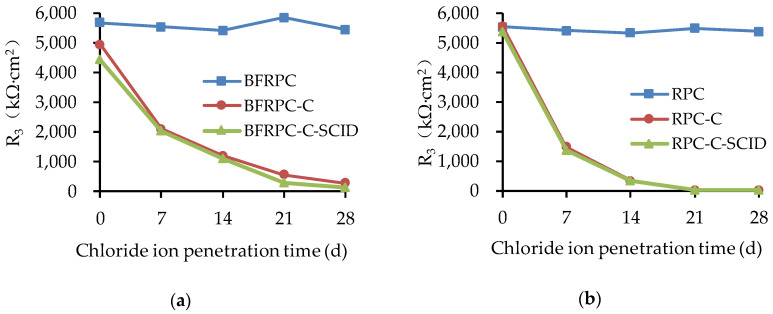
Polarization resistance R_3_ for: (**a**) BFRPC specimens; (**b**) RPC specimens.

The results of the resistance (R_3_) of the BFRPC specimens and RPC specimens are given in Figure 11.

Figure 11 indicates that the steel resistance in the specimens without cracks has little change with the increase of chloride ion penetration time, which is about 5000–6000 kΩ. This elaborates that the steel bars are not corroded, which is consistent with the results of E_corr_ and i_corr_. However, the R_3_ of BFRPC-C, BFRPC-C-SCID, RPC-C, and RPC-C-SCID decreases with the increase of chloride ion penetration time. It can be inferred that cracks have a great influence on the corrosion of steel bars embedded in BFRPC. Compared with the uncracked area, the steel bars area at the crack is not protected by the concrete matrix, and the active corrosion will occur first. The crack increases the overall corrosion activity of the steel bars at the crack. Furthermore, it can be seen from Figure 11 that SCID has less effect on the resistance of steel bars embedded in BFRPC than the crack. This is because of BFRPC’s excellent compactness and BF bridging function, so the corrosion activity at the crack is not suitable for propagation, and it is not easy to reach the SCID position and cause corrosion diffusion.

### 4.3. Discussion

**Figure 12:**
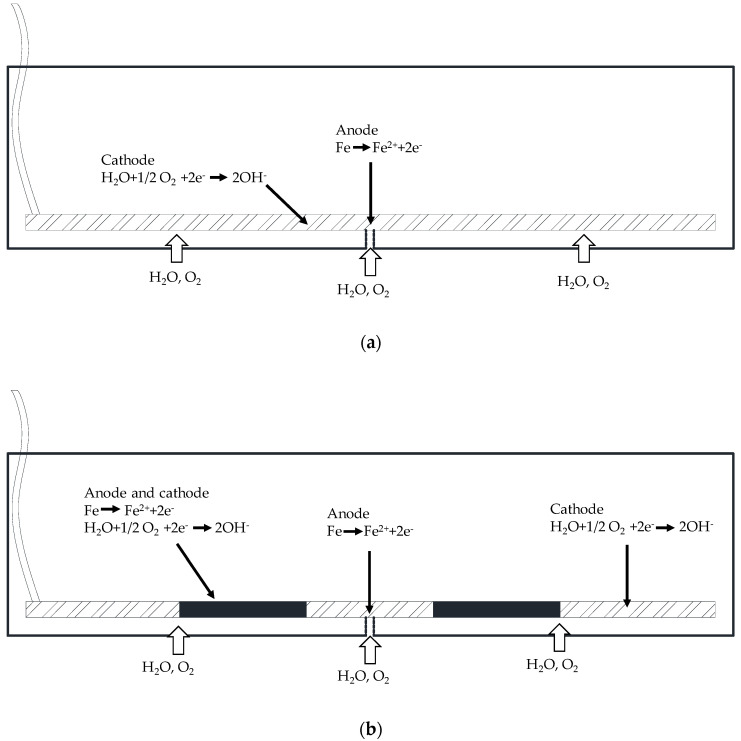
Corrosion mechanism of steel in conventional concrete: (**a**) crack, (**b**) crack and SCID.

Hay et al. [23] found that the steel bar at the crack location in conventional concrete would preferentially undergo active corrosion due to the absence of concrete matrix protection. As shown in Figure 12a, the steel in the vicinity of cracks acts as the anode, while the steel embedded in the concrete around it acts as the cathode, resulting in the gradual extension of the corrosion along the steel bars. Figure 12b elaborates that when the SCID exists between steel bars and concrete, steel corrosion is no longer confined to the crack location and will be associated with corrosion at the SCID location, further aggravating steel corrosion. Therefore, crack and SCID have a great influence on the steel corrosion of conventional concrete [36].

In this paper, the effects of cracks and SCID on steel corrosion in reinforced BFRPC were studied. The E_corr_ and i_corr_ values of the steel bars in the specimens with cracks both exceeded the threshold value of steel corrosion, compared with the specimens without cracks. This was consistent with Hay’s findings [23], suggesting that cracks have a similar effect on steel corrosion in BFRPC and conventional concrete. Cracks had a great influence on the steel corrosion of reinforced BFRPC, so the occurrence of cracks should be controlled in practical engineering.

Under the action of mechanical load, a certain degree of slippage and separation is easy to occur between steel bars and concrete matrix, resulting in SCID [43]. The above SCID is characterized by interface microcracks, while the filter paper in this paper simulates the SCID characterized by a porous interface [44]. The inherent steel-concrete interface conditions, such as concrete bleeding, segregation, and settlement of newly mixed concrete will produce porous SCID [45]. The SCID simulated by filter paper is mainly to reflect the exfoliation caused by high mechanical load or bleeding. Such porous SCID will cause early corrosion, which has a great negative impact on the corrosion of steel bars in concrete [46]. In this paper, the effect of SCID on steel corrosion of BFRPC was somewhat different from the found in ordinary concrete in previous studies [23]. The experimental results revealed that the effect of SCID on the E_corr_ and i_corr_ values of steel bar in reinforced BFRPC were smaller. This phenomenon indicated that the steel corrosion only exists in the vicinity of the crack location in reinforced BFRPC, rather than connected to SCID locations. Many researchers [47,48,49] had confirmed that RPC had very high compactness. The dense RPC matrix prevented the water and oxygen from reaching the steel bar location, thus preventing the SCID from aggravating the steel corrosion. Therefore, the influence of SCID on the steel corrosion of reinforced BFRPC could be ignored in engineering.

As for the influence of BF on the steel corrosion, this paper mainly studied the influence of BF on the corrosion propagation of steel bars. A large crack width (1.5 mm) was selected to eliminate the effect of the crack geometry and the bridging fiber between the cracks on the steel corrosion. In the study, the crack width in the specimens of BFRPC-C and RPC-C remained unchanged, so the corrosion activity of the steel bars in the specimens of BFRPC-C and RPC-C would mainly depend on the BF. This study showed that the corrosion activity of BFRPC-C was less than that of the control group. Crack and SCID lead to early initiation of corrosion damage of steel bar embedded in BFRPC and RPC. After the steel corrosion, the gradual increment of corrosion products would lead to microcracks initiation in concrete [23]. The bridging action of BF increased the mechanical properties of RPC and the cracking resistance caused by corrosion products. Therefore, BF prevented the propagation of corrosion though the whole steel bars and improved the corrosion resistance of RPC.

## 5. Conclusions

In this study, the steel corrosion behaviors of reinforced BFRPC material under the effects of cracks and SCID were studied by the electrochemical measurements, and the beneficial role of BF on controlling steel corrosion was investigated. Several conclusions derived based on the experimental results in this study may be summarized as follows:

(1) Cracks have a great influence on the steel corrosion of reinforced BFRPC, so the occurrence of cracks should be controlled in engineering.
(2) BFRPC is different from conventional concrete due to its excellent compactness, preventing water and oxygen from reaching the steel bar location and preventing the adverse effects of SCID.
(3) BF can effectively control the cracks generated by the expansion of corrosion products and extend the in-service life of the reinforced BFRPC.

The study clarified, in a controlled manner, the effects of cracks and SCID on steel corrosion in reinforced BFRPC, and demonstrated the beneficial role of BF in corrosion control. It provided a reference for the popularity of BFRPC in practical engineering.

## Figures and Tables

**Table 1:** Basic properties of basalt fibers.

Fiber Type	Length (mm)	Diameter (μm)	Linear Density (tex)	Tensile Strength (MPa)	Elastic Modulus (GPa)	Breaking Strength (N/tex)	Elongation (%)
Basalt fiber	22	23	2392	2836	62	0.69	3

**Table 2:** Mix proportions of RPC and BFRPC (kg/m^3^).

	Water	Cement	Silica	Quartz Sand		Quartz Powder	Basalt Fiber	Water
			Fume	0.15 mm–0.3 mm	0.3 mm–0.6 mm			Reducer
BFRPC	151.5	841.8	210.4	364.2	582.8	311.4	12	52.6
RPC	151.5	841.8	210.4	364.2	582.8	311.4	0	52.6

**Table 3:** Description of specimen conditions.

Specimen Types	Crack	Steel-Concrete Interface Damage (SCID)
BFRPC; RPC	○	○
BFRPC-C; RPC-C	●	○
BFRPC-C-SCID; RPC-C-SCID	●	●

The “○” in the table indicates that the item is not included, and “●” indicates that the item is included.

**Table 4:** Electrochemical parameters of the Tafel curve.

Specimen Number	b_a_ (mV/Decade)	b_c_ (mV/Decade)	i_corr_ (μA/cm^2^)	E_corr_ (V)
BFRPC	371.580	159.118	0.042	−0.146
BFRPC-C	471.483	397.299	0.545	−0.563
BFRPC-C-SCID	678.020	295.855	0.357	−0.609
RPC	257.130	233.506	0.029	−0.248
RPC-C	504.852	188.842	0.492	−0.711
RPC-C-SCID	635.045	525.975	0.848	−0.678

**Table 5:** Electrochemical parameters of EIS test.

	R_1_ (Ω·cm^2^)	CPE_1_-T (μF·cm^2^)	CPE_1_-P (μF·cm^2^)	R_2_ (Ω·cm^2^)	CPE_2_-T (μF·cm^2^)	CPE_2_-P (μF·cm^2^)	R_3_ (kΩ·cm^2^)
BFRPC	175.0	5.139 × 10^−^^8^	1.22	4702	3.45 × 10^−^^7^	0.57	5093
BFRPC-C	145.9	2.434 × 10^−^^8^	0.79	3604	4.71 × 10^−^^5^	0.43	279.55
BFRPC-C-SCID	134.6	2.48 × 10^−^^8^	0.78	3654	4.60 × 10^−^^5^	0.44	114.49
RPC	203.2	9.23 × 10^−^^8^	1.05	2836	3.24 × 10^−^^7^	0.63	2531
RPC-C	138.0	2.67 × 10^−^^8^	0.89	942	3.57 × 10^−^^5^	0.72	40.81
RPC-C-SCID	155.2	3.24 × 10^−^^8^	0.87	1027	3.61 × 10^−^^5^	0.74	24.94
